# Differentiating authentic versus pseudo vulnerability in therapeutic practice^1^

**DOI:** 10.3389/fpsyt.2023.1200981

**Published:** 2023-10-30

**Authors:** Susan G. Simpson, Limor Navot

**Affiliations:** ^1^UniSA Justice & Society, University of South Australia, Adelaide, SA, Australia; ^2^NHS Forth Valley, Larbert, United Kingdom; ^3^SafePath Institute, Epen, Netherlands; ^4^U-center, In-Patient Clinic, Epen, Netherlands

**Keywords:** Schema Therapy, authenticity, schema modes, pseudo vulnerability, Helpless Surrenderer mode, Complaining Protector mode, Self-Pity/Victim mode

## Abstract

The importance of empathy and attuned care as key ingredients in therapeutic healing has been widely recognized. However, empathy that is delivered in ways that are misaligned with the client’s clinical presentation and emotional states or ‘modes’ can have the opposite effect, reinforcing unhelpful coping patterns, and hindering recovery. In this theoretical development paper, it is proposed that ‘pseudo vulnerability’ is an important yet overlooked source of therapeutic impasses, often resulting in unremitting clinical problems, and poor clinical outcomes. A range of commonly occurring pseudo vulnerable presentations are described, including Complaining Protector, Attention/Recognition Seeking, and Self-Pity/Victim, with the addition of a new mode Helpless Surrenderer. Guidance on differentiating pseudo vulnerable modes from each other and from the authentic Vulnerable Child mode are delineated via characteristic clinical presentations and typical therapist countertransference reactions. Methods for managing pseudo vulnerability to facilitate access to underlying authentic vulnerability are described.

## Introduction

The capacity to respond to clients’ distress and helplessness with empathy and care is arguably the lynchpin of sound therapeutic practice. Indeed, the therapist’s ability to convey empathy is widely considered a core feature of an authentic therapeutic alliance, and the foundation for effective psychotherapy outcomes (1, 2). While these are core skills that are taught and considered essential across psychotherapy training programs (3, 4), what happens when standard responses of empathy and warmth do not help, or worse still, appear to perpetuate or exacerbate the clients’ difficulties or psychopathology? In spite of psychotherapists’ best efforts to work with clients who present with high levels of vulnerability, hopelessness and helplessness, it is not uncommon to experience ‘stuckness’ and a sense of frustration that little progress is being made (5, 6, 7). For clients, this internal sense of ‘Groundhog Day’ can reinforce their experience of helplessness and hopelessness, their perception of ‘victimhood’, as well as dependence on a system which appears to be ineffective in meeting their needs. In turn, this can impact therapists’ own sense of competence and self-efficacy, fueling a sense of helplessness, as well as a sense of guilt that they are failing their clients. These factors not only impact negatively on client motivation but also increase risk of therapist burnout (8, 9).

In this article, we argue that ‘pseudo vulnerability’ is an important and overlooked reason that therapy can become stuck, resulting in long-term yet ineffective episodes of care in therapeutic settings. A few authors have called attention to some of these pseudo vulnerable self-states or ‘modes’ in the lexicon of Schema Therapy (10), including the Complaining Protector mode (11), and Victim/Self-Pity mode (12, 13). In this article, we argue that pseudo vulnerability is the central theme that these modes share in common, and is a concept warranting further attention. Moreover, we present several different mode variants involving pseudo vulnerability, including some described here for the first time. We describe these modes phenomenologically, explain the maladaptive coping mechanisms involved, discuss the challenges they pose, including therapists’ reactions to them, and techniques to overcome them. Thus, our goal is to provide a unified framework, based on the theoretical model of Schema Therapy, to help therapists recognize, understand, and intervene effectively with them.

### Context: a Schema Therapy perspective

Over past decades, a range of process-oriented therapeutic models have been developed to address more complex psychological difficulties that do not respond to maintenance model psychotherapies (i.e., brief models that focus solely on here-and-now manifestations of the problem). Schema Therapy is one such model, specifically designed to address deeper level emotional difficulties, with a well-established evidence base for the treatment of a range of complex clinical presentations (10). According to the Schema Therapy model, the interaction of temperament with consistently unmet attachment needs is associated with the development of early maladaptive schemas. These operate as predictive templates through which we view the world and interpret experience.

The here-and-now state-based manifestation of schemas are referred to as ‘modes’ (10). Modes refer to states of mind that together constitute the ‘self’. In a healthy self-system these modes are integrated, operating in a flexible manner which maximizes allostasis. In contrast, in the face of adverse childhood experiences, modes can become dissociated and operate in an overly rigid or chaotic manner. Four main types of modes have been proposed. (1) The Child [emotional] modes represent the feeling states that we are all born with, and which alert us to our emotional needs. When emotional needs are not met adequately and consistently, it is the Vulnerable Child mode that experiences high levels of distress and overwhelm (“I feel sad, anxious, lost”). (2) The Introjected modes represent internalized critical, guilt-inducing or demanding messages that we receive from caregivers, society, educators, peers, and so on, and operate as intrusive Inner-Critic ‘voices’ from the past that echo perseveratively within the person’s mind (e.g., “You’re useless”). (3) The Healthy Adult mode is a flexible, integrated inner leader which is able to self-reflect and behave in a manner which balances one’s own needs with those of others. This mode acts as an internal compassionate leader that coordinates all of the other internal modes in a manner which promotes homeostasis. (4) The Maladaptive Coping modes are the parts of self that develop in childhood or adolescence to protect the person from overwhelming distress, thereby enabling them to survive when childhood needs are not met. Three main types of coping modes have been identified in Schema Therapy: (1) The Avoidant modes (e.g., dissociation, emotional or behavioral avoidance, impulsive or compulsive self-soothing); (2) The Compliant Surrender mode (e.g., compliance, subservience, obedience); and (3) The Overcompensatory modes (e.g., striving to be superior, perfect, hyper-autonomous, powerful 14, 15). Most coping modes are formed in childhood and adolescence, and therefore represent the part of the child that has implicitly overlearned coping mechanisms that have become entrenched and automatic, persisting into adulthood. It has been suggested that each mode consists of a set of distinct features, including affect, behaviors and cognitions, which are synchronously activated for distinct periods of time (16).

In Schema Therapy, the goal is to stimulate the client’s natural emotional progression from wherever they have become developmentally ‘frozen’ as a result of unmet emotional needs. Through a process of *Limited Reparenting*, the therapist attempts to connect with the client’s vulnerability (i.e., their Vulnerable Child mode) in order to provide some of their core unmet needs as an antidote to missing attachment needs in childhood. In this way, the therapist’s reparenting messages become internalized and form a foundation for the client’s own Healthy Adult mode. Over the course of therapy, the therapist transitions to coaching the client’s own Healthy Adult mode to take responsibility for their own wellbeing, thereby facilitating healthy individuation, and autonomy. The ultimate goal is for the client to reach emotional maturity, with a self-compassionate Healthy Adult self that is ‘at the helm’ of their inner mode system. Limited re-parenting typically includes, among other elements, a balance of nurturance, attunement, empathy, and emotional guidance. It also includes encouragement to assert needs, alongside support with learning to respect others’ needs and boundaries through limit setting and frustration tolerance. The re-parenting ‘recipe’ is determined by a detailed assessment and case conceptualization, but the way it is delivered is to a large extent determined by the ways in which the client’s modes manifest within sessions. The schema therapist’s goal is to bypass the coping modes via empathic confrontation and limit setting, in order to connect with the client’s genuine vulnerability (i.e., Vulnerable Child mode). In this way, the early emotional wounds of the client can be healed. However, this process can be derailed when the conceptualization becomes confounded by apparent manifestations of vulnerability that are in fact just another coping mode designed to cleverly ‘mask’ and distance others from an individual’s true vulnerability.

### The missing link in Schema Therapy conceptualization

We propose that *pseudo vulnerable coping modes,* which either resemble or mimic the Vulnerable Child mode, represent a missing link within the standard Schema Therapy conceptualization. Whereas pseudo vulnerable modes are characterized by outward manifestations of high expressed emotion and/or complaint, further questioning often reveals the person has little if any genuine connection to core emotions. Pseudo vulnerable modes consist of vague or diffuse global distress, which are manifested as states of helplessness, hopelessness, self-pity, or compliance. Indeed, this is consistent with previous descriptions of ‘global distress’ in the context of Emotion-Focused Therapy, as an “undifferentiated emotional response characterized by high arousal and low meaning” [(17), p. 61]. These states are in direct contrast to the differentiated states of the Vulnerable Child mode that are closely tied to schemas and unmet needs (18).

Mistaking a maladaptive coping mode for the Vulnerable Child mode can lead therapists to unwittingly provide nurturance when what is actually required is empathic confrontation, thereby reinforcing unhelpful ways of coping. This process of inadvertently reparenting a coping mode, commonly leads to extended therapy episodes with little progress. We suggest that when clients present in therapy in pseudo vulnerable modes, their capacity to self-reflect and to carry out emotional processing is blocked. Consequently, limited reparenting of a pseudo vulnerable mode can be iatrogenic, fostering an unhealthy level of dependence on the therapist with minimal if any lasting change. In other words, when therapists try to provide empathy or nurturance to clients who are presenting with pseudo vulnerability, they may be surprised at the results. Rather than calming, soothing, or comforting the client, or helping them to heal their pain, they might find that clients only persist in their distress and dependence on the therapist. Ultimately these modes can and frequently do block opportunities to stimulate authentic emotional connection and growth (19).

Clinical experience suggests that many clients who present with pseudo vulnerable modes have had minimal opportunities in life to ‘taste’ authentic emotional closeness. They may never have had the opportunity to share their genuine vulnerability with others, nor to experience emotional presence, soothing, mirroring, or deep attunement within primary relationships. As a result, they have ‘given up’ hope for attuned care (20). Instead, in early attachment relationships, genuine care and attunement may have been demonstrated through ‘pseudo-connection’, consisting only of practical ‘solutions’ for their pain, ‘rescue’, or pity. As these ingredients represent the closest approximation to intimacy that the client has experienced in their close relationships, these have become the ingredients they consistently seek, in place of authentic emotional connection and intimacy (21). However, these ingredients can never fully satisfy the underlying longing for authentic connection, and the client is ultimately thwarted by competing yearnings for and fears of intimacy. When there is no template for true intimacy, even when these ingredients are available within adult relationships, the person is often unable to recognize, or ‘digest’ them. Within the therapeutic relationship, the countertransferential experience is that the therapist experiences themselves as a ‘fix’ that is being ‘used’, rather than a separate emotional entity, a real living person, available for authentic connection.

We suggest that when therapists misconstrue pseudo vulnerable modes as genuine vulnerability (i.e., Vulnerable Child mode or Angry Child mode), they are more likely to employ ineffective therapeutic interventions that perpetuate these maladaptive coping mechanisms. Further, therapists may unwittingly find themselves drawn into taking a ‘rescue’ stance, holding back from employing their most powerful healing methods due to fears of destabilizing the client, and instead resorting to a pattern of providing an endless supply of strategies, solutions and support. This dynamic has previously been explored in psychoanalytic writing, whereby the rescuer phenomenon is well-recognized as a manifestation of therapeutic countertransference. Berman (22), refers to discussion of this by Freud, elaborated by Ferenczi in a 1919 paper where he describes situations in which “the doctor has unconsciously made himself his patient’s patron or knight,” and recognized that he had himself fallen into this trap [(22), p. 429]. Therapists with a self-sacrifice schema (belief that they are responsible for meeting the needs of others at the expense of their own gratification) may be particularly prone to taking on the rescuer stance (23).

This dynamic was further elaborated by Karpman (24) through the Drama Triangle concept within Transactional Analysis. According to this perspective, individuals tend to assume one of three main roles within their family of origin: (1) The ‘Victim’ believes they are powerless to change their difficult circumstances, and that in spite of their efforts, nothing makes a difference. They view others either as potential persecutors, or saviors who can solve their problems. (2) The ‘Perpetrator’ blames others for their problems, and may be controlling, angry, taking a ‘one-up’ stance; (3) The ‘Rescuer’ focuses on helping others in order bolster their own self-worth and to alleviate unresolved feelings of guilt. From time to time, individuals may switch out of their default roles, for example the Rescuer may become resentful and switch into Persecutor, or the Victim may feel guilty and burdensome, switching into Rescuer. In all three positions, the focus on blaming, rescuing or help-seeking can become entrenched as the standard modus operandi through which the person manages relationships and avoids facing their own underlying insecurities. Within the Schema Therapy model, all three of these positions represent Maladaptive Coping modes, and the key to a healthy interpersonal dynamic lies in building the Healthy Adult mode which can connect to the authentic Vulnerable Child mode.

When therapists are unable to differentiate the pseudo vulnerable modes described in this paper [that fall within the Victim position on the Drama Triangle], they will be more prone to blindly acting out the countertransference through taking on the Rescuer role. Other times, this dynamic may inhibit proper therapeutic interventions. For example, the therapist may feel afraid of confronting the client (in the Victim role) due to fears of being identified with the Perpetrator. In turn, clients can become increasingly entrenched in patterns that take them further from recovery, for example, looking for the therapist to ‘save’ them, feeling helpless or hopeless, constantly complaining, or simply seeking sympathy. Ultimately, the pseudo vulnerable coping modes can passively take control over the entire therapy process, interfering with the therapist’s capacity to mentalize, to set healthy boundaries and adhere to the Schema Therapy treatment model. The harder the therapist works to reparent a client in a pseudo vulnerable coping mode through nurturance and attunement, the more stuck the therapy process ultimately becomes. The bigger picture is that, if not recognized and managed accordingly, the pseudo vulnerable modes can lead to more frequent and extended (largely ineffective) in-patient admissions, as well as longer outpatient episodes within mental health services from as early as childhood.

### Working with pseudo vulnerable modes

In this article, we provide case descriptions and illustrations based on pseudo vulnerable coping modes. Mode presentations do not necessarily appear in the pure forms described in the literature, and the specific sequences in which they manifest can be highly idiosyncratic (23), and may manifest as causal networks that mutually impact on each other (e.g., Abandoned Child triggers Inner Critic, which in turn activates the Helpless Surrenderer) (25) Therefore, the case examples provided here are not intended to be demonstrations of best practice in Schema Therapy, nor as complete case conceptualizations. They are illustrations of common prototypes of the proposed pseudo vulnerable modes and the common countertransference reactions to them. In our clinical experience, these modes require a specific approach to address the underlying coping function. This requires therapists to become mindful of acting out countertransference action tendencies, which can lead to misinformed and potentially iatrogenic clinical decision-making (26, 27). Due to the powerful yet passive interpersonal dynamics employed by pseudo vulnerable coping modes, and their tendency to operate ‘under-cover’ out of conscious awareness, we suggest that skillful mode work is likely to be more effective than directly targeting the schemas. It is only when the therapist bypasses the coping mode to reach the underlying authentic Vulnerable Child mode that the schemas can be effectively healed.

In the next sections we will first describe the presentation and function of several versions of coping modes that present with pseudo vulnerability (i.e., Complaining Protector, Attention/Recognition Seeking, and Self-Pity/Victim) including a new mode (i.e., Helpless Surrenderer mode). Further, we will describe common countertransference reactions to these modes, and suggest ways of differentiating pseudo vulnerable modes from each other and from the authentic Vulnerable Child mode. Finally, we provide steps for working with pseudo vulnerable modes.

## Brief description of pseudo vulnerable modes: surrender, avoidant, overcompensatory

### Helpless Surrenderer

The Helpless Surrenderer is a surrendering coping mode that both seeks to avoid rejection and abandonment and to seek out attachment needs through passive and indirect forms of expression (14, 21). The client presents as helpless, with the expectation that the therapist will provide solutions through quick-fix practical strategies. In this mode the person is seeking to be rescued by others who are expected to solve their emotional difficulties. Here we describe two different versions: Care seeking, whereby the person actively attempts to communicate attachment needs in a clingy-dependent and often covertly entitled manner; and Resigned, whereby the needs are suppressed and manifested via the body, often in a partially dissociated manner.

- *Helpless Surrenderer (Careseeker)*: manifests as care seeking or care-eliciting behavior through passive, helpless, and often frantic begging and pleading. This mode presents as regressed, clingy, and emotionally (or physically) fragile. The person may hint at risky behavior or suicidality as a means of seeking care. In this mode the client wants to be ‘saved’, or ‘rescued’, yet no amount of care appears to allay the global feelings of distress.
- *Helpless Surrenderer (Resigned)*: manifests as hopeless, frozen, misunderstood, fearful of individuation, and avoidant of adult responsibilities. In this mode, emotional needs are mostly communicated and made visible to others via physical fragility, that conveys helplessness and vulnerability. Medical symptoms/illness, somatisation and conversion symptoms may also operate as manifestations of this mode.

### Complaining Protector

The Complaining Protector is an avoidant coping mode that uses a ‘wall of complaints’ to keep others away from the person’s painful emotions. Here we describe two different versions of this mode:

- *Complaining Protector (Help-seeking-help-rejecting):* complains about the person’s suffering and invites people to listen, but under the assumption that problems will not be solved. This mode is not looking for a real solution or genuine advice as it repeatedly rejects any offers for help.
- *Belligerent Complaining Protector:* involves a main behavioral pattern of complaining, but with the main focus on *others’ wrongdoings.* The purpose of this mode is to find more and more reasons to complain about how others, for example, are not doing their job, not behaving appropriately, or are mistreating the person.

### Attention/Recognition Seeking (pseudo vulnerable version): ‘Dont blame me’

The Attention-Seeking mode presents with exaggerated and superficial displays of emotionality. There is usually a mismatch between the content of what the person is saying and how it feels to the observer. A version of this mode can also present with pseudo vulnerability, as the person presents with a sudden, intense, or highly dramatic manifestation of pain and suffering. The function is to draw the attention to the person and his suffering, but it may also represent an unconscious desire to evade responsibility or blame. This mode is often accompanied by a theme of ‘victimhood’. The intensity of the victim presentation often ‘neutralizes’ others from criticizing or confronting the person.

### Self Aggrandizer (pseudo vulnerable version): ‘Self-Pity/Victim’

The Self-Aggrandizer mode often involves acting in a manner that is dominant and superior, demeaning others, as a means to achieving power and success. The covert version of Self-Aggrandizer, Self-Pity/Victim, can also present with pseudo vulnerability, whereby the presentation of ‘suffering’ comes across as superficial, singing to the tune of *‘poor me, I am sacrificing so much, but I have no choice in the matter’.* This person will often present their suffering in relation to his tendency to be a martyr or saviour or a self-sacrificer, whereby suffering is worn as a ‘badge of honour’.

For an overview of pseudo vulnerable modes, and associated presentations, unmet needs and countertransference reactions, see Table 1.

**Table 1:** Description of Pseudo Vulnerable modes.

**Pseudo vulnerable modes**	**Presentation**	**Language**	**Function**	**Common countertransference reactions**
**Helpless Surrenderer**	**‘Helpless Careseeker ‘** Fantasises about being adopted, saved, rescued. Clinging, reassurance-seeking, hinting at or acting out risk/ suicidality, ‘collecting’ diagnoses.	*‘I can’t do it, it’s too much for me’; ‘That didn’t work, I already tried that’,* *‘I’m too fragile to manage that’* *‘You don’t really care about me if you don't give me the help I need”’*	To fulfil the fantasy of accessing the ‘perfect care’ that was missed in childhood. To avoid the grief of facing the reality of childhood neglect. To *prove* or *show* deservingness of care and/or guilt-induce others to provide care.	Stage 1: Feeling sympathy, compassion, care, urge to rescue/fix/save. Frustration with colleagues who do not engage with the client. Urge to collude through avoiding setting limits and discussing therapeutic endings. Stage 2: Feeling stuck, inadequate, trapped, hopeless, helpless, irritated, burned out. Feeling guilty for not doing enough and waning feelings of compassion.
	**‘Helpless Resigned’** Seeks reassurance, advice. Converts emotions and needs into physical ‘symptoms’. Physical illness, fragility, pain, manifest as a metaphor for ‘unspeakable’ vulnerability and needs.	*“Fix me, I can’t do it”. “What shall I do now?” “That didn’t work, what else?” “I wish I could go to sleep and when I wake up it's all fixed”.*	To express vulnerability and need for connection in ways considered legitimate by significant others. To bypass guilt/shame associated with direct expression of needs via conversion into ‘concrete’ symptoms and illness. To seek solutions to be ‘fixed’ whilst avoiding facing painful emotions.	Feeling stuck, hopeless, helpless, frustrated, confused, powerless. Urge to provide reassurance, solutions, strategies.
**Complaining Protector**	**‘Help-seeking-help-rejecting’** Complains and invites people to listen, with the underlying assumption that problems will not be solved. The person is not looking for a real solution or genuine advice. The emotional presentation is mostly ‘flat” although in some cases may involve slight annoyance or frustration.	*“I am suffering because of so many things. Listen to my complaints, but I won’t let you help me”.*	To avoid painful emotions by creating a ‘wall of complaints’.	Feeling emotionally ‘numb’ and trying to think rationally about possible solutions; Feeling a sense of pressure to come up with the ‘right’ solution to the problem; Feeling frustrated or a sense of ‘failure’.
	**‘Belligerent Complaining Protector’** Complains with the main focus on *others’ wrongdoings.* There is hyper-focus on external detail. Others’ behaviors are perceived as the main cause of the person’s suffering. The emotional presentation often involves surface-level frustration or agitation, but the underlying rage and anger are not fully expressed.	*“Others are not doing their job, not behaving appropriately, or mistreating me”;* *“I am a victim of circumstances. Others need to change”.*	To avoid closeness and genuine expression of emotions by focusing on others’ wrongdoing and blaming others.	Feeling companionate toward the client; Colluding with this mode by perceiving the person as the ‘victim’ and others as ‘harmful’. Feeling the need to ‘save’ the client. Feeling frustrated, angry, or helpless.
**Attention/** **Approval Seeker:** ***‘Don't blame me’***	Displays intense ‘vulnerability’ in ‘dramatic’ ways as a response to confrontation, or when the person knows he is about to be confronted or held accountable.	*“I am suffering - don’t confront me”; “I am not accountable because of my emotional pain” / “Don’t confront me because I am already blaming myself / Guilt-inducing messages: Look what you did to me - you made me suffer more!”*	To take the ‘one up’ position by drawing the focus to the person’s suffering; To evade responsibility or blame by ‘neutralizing’ others (i.e. using vulnerability as power). To induce guilt in others in order to protect oneself from criticism.	‘Walking on eggshells’ when around the client. Feeling shocked and overwhelmed. Feeling guilty, ashamed, or ‘inadequate’. Feeling angry or enraged.
**Self-Aggrandizer: ‘Self-Pity/Victim’**	The presentation of ‘suffering’ comes across as superficial, and involves a theme of ‘poor me’. The suffering is often associated with the person’s tendency to ‘self-sacrifice’, or to be a ‘saviour’ or a ‘martyr’. The behavioural presentation often includes moaning, wailing, showing tiredness and exhaustion, or rolling eyes.	*“Poor me, I am suffering so much”; “I am doing so much for others, I have to make sacrifices, but I have no choice in the matter!”*	Uses suffering to seek pity, recognition, or admiration. Seeks self-importance, meaning, or control by presenting self as a ‘martyr’.	Irritated, angry, guilty (for not feeling more empathic).

## Pseudo vulnerable modes- key clinical features

### Surrender pseudo vulnerable modes

#### Helpless Surrenderer mode

The Helpless Surrenderer presents as a mode that overtly or covertly seeks to be rescued or ‘saved’ via indirect, or passive attempts at ‘showing’ vulnerability and stuckness. This may be either through dependent-entitled care seeking and/or via bodily symptoms, illness and physical frailty. In therapy sessions, this mode may manifest in more extreme forms such as overt pleading, begging and reassurance seeking, taking on the sick role such as through seeking out diagnoses, or more subtle manifestations such as via medically unexplained pain or low weight. The Helpless Surrenderer develops as a result of implicit overlearned experiences in childhood, whereby normal direct expressions of distress and care seeking have led to disappointment, shame and guilt-inducing responses from others. The child is left with a dilemma in terms of how to find connection in a way that *shows* their distress in a manner that others will view as worthy of a response. The Helpless Surrenderer emerges from this paradox, as an attempt to communicate distress through whatever means is deemed as legitimate and deserving in the family of origin.

In Helpless Surrenderer mode, the mindset is aligned with the medical model, whereby diagnoses can become the legitimizing proof of the person’s helplessness and deservingness of care. The therapist feels compelled to generate solutions, with the client as the passive recipient. From the client’s perspective, the success of therapy is viewed according to the therapist’s capacity to generate strategies that bring immediate relief from distress and difficulties. Therapists commonly become stuck in an endless rut of working harder than the client, reparenting through empathy, care and attempting to generate better ‘strategies’ that will help the client. Instead of healing, standard reparenting operates as a form of ‘collusion’ with the coping mode. Although this may provide short-term relief, this pattern ultimately reinforces and gratifies this mode. This pattern can be powerfully played out in in-patient wards, which provide the ultimate environment for ‘saving’ the client and providing the care they have been longing for. In Helpless Surrenderer mode, this may manifest through risk- or illness-behaviors that increase chances of remaining in the ‘safe haven’ of the hospital environment, while outwardly protesting that they wish to be discharged.

Alongside all of the pseudo vulnerable modes described here, according to Edwards (23) the Helpless Surrenderer may manifest as a blend or as part of a sequence of modes. For example, Edwards describes a range of specific manifestations of coping modes such as the ‘Care Seeking (Secondary Gain) Overcompensator’ and the ‘Hypervigilant Clinger’ that may manifest as one or more of a blend of modes under the broad umbrella of the Helpless Surrenderer (Care Seeker) mode. Recognizing these mode-variations may help to fine-tune our understanding of the range of specific manifestations or ‘flavours’ of the Helpless Surrenderer mode.

##### Helpless Surrenderer (‘Careseeker’ version)

This version of the mode more often manifests with clients with anxious-ambivalent attachment and dysregulated/undercontrolled difficulties, including Cluster B personality traits.

The Helpless Surrenderer (Careseeker) is ultimately a mode that is seeking care and connection through helpless coping behavior. This mode presents in sessions as dependent, incapable, feeble, teetering on the edge of emotional collapse. Overt expressions of distress and suffering are described in an intense, helpless manner. This mode can present as clingy and entitled, begging, pleading, and whatever the therapist offers in terms of reparenting never quite feels enough. This mode can present as frustrated and entitled, based on the expectation that the therapist *should* be providing more care. Common cognitions include: ‘I cannot do it…I need you to do it for me.’ ‘If only I can show others how lost and hurt I am, then they will take care of me.’ They may also feel resentful, victimized, and envious of others, especially the therapist’s family and other clients.

A client in this mode may talk about their suffering through the use of global diagnostic terminology to describe vague pseudo emotional states (e.g., “My panic attacks are worse” “I’m suicidal”), the sick role (e.g., “I collapsed earlier this week”) and/or thinly veiled hints at risk (e.g., “Do not make me talk about the past…it gives me suicidal thoughts”). This can function as a means of eliciting pseudo connection and care whilst avoiding the authentic connection that may put them in touch with underlying core emotions. Over time, the person can become so accustomed to operating from this mode that it becomes a core part of their identity. The type of care that is sought tends to be aligned with the type of care that was available within the person’s family of origin – whether it be ‘solutions’ in the form of practical advice, reassurance, skills, or sympathy. However, the relief that these provide is generally short-lived, both because these types of care tend to be superficial and unsatisfying, but also because the Helpless Surrenderer (Careseeker) is not the authentic Vulnerable child, and therefore the care that is provided is not ‘absorbed’, but rather operates as a short term ‘fix’, followed by a demand for more.

The inability to absorb care can lead to chronic dissatisfaction, and a sense that whatever is offered is never enough. The reparenting relationship comes to function more as an ‘external-emotional-regulator’ which is never fully internalized and incorporated into the person’s own Healthy Adult. In the person’s attempt to be saved or rescued the potential for true intimacy and authentic connection are sacrificed.

The frantic search for a rescue figure enables the client to prevent the pain of the past and the memories of abandonment and neglect (i.e., “If I can find a new parent-substitute, I do not need to face the pain, grief and anger of what happened to me”). When directed at this coping mode, empathic messages provide only short-term relief, resulting in an unquenchable craving for more. The urgency with which the Helpless Surrenderer (Careseeker) seeks a perfect rescuer or nurturer therefore hampers the person’s capacity to absorb and internalize reparenting messages in any sustained way, thus thwarting the development of object permanence. As a result, the therapist can find themselves working ever harder in order to fill what seems like a bottomless pit of need. The therapist is recruited as the person who must save the client or make the suffering disappear by providing ‘perfect’ care. However, the demanding and coercive nature of attachment seeking from this mode ultimately results in a self-fulfilling prophecy whereby the person repeatedly relives the same interpersonal drama, leading others to withdraw due to feeling alienated, burned-out, and frustrated.

In this mode, the person may pay lip-service to therapeutic goals but has the underlying survival agenda of seeking a ‘perfect’ nurturer who can meet all of their needs and fulfill their childhood fantasy of being rescued. There is a tendency to view the therapist as an idealized ‘knight in shining armour’, who holds the keys to solving their difficulties. Therefore, when the therapist confronts this mode, the person may shift into another pseudo vulnerable mode with a victim stance “You do not care about me”, with the function of maneuvering the therapist back into a more sympathetic rescue position. Attempts by the therapist to use usual experiential techniques such as Imagery Rescripting can become an opportunity for the Helpless Surrenderer to either re-live their rescue fantasy rather than emotional processing, or to avoid altogether (e.g., “I cannot do imagery…I was so overwhelmed last time that I could not cope”). Attempts by the therapist to discuss termination of therapy tend to be met with increased acting-out behaviors (e.g., risky behaviors, self-harm, weight loss), and/or guilt inducing messages (“You’re abandoning me.you do not care about me”).

Helpless Surrenderer (Careseeker) is illustrated in the following case example:

**Table:** 

Serena a 25-year-old woman, presented with depression and borderline personality disorder. Relationships followed a pattern of intense closeness, followed by a period whereby others distanced themselves. Serena presented as distressed and helpless in sessions. When invited to describe her emotions, she wept silently, or described symptoms or global distress (‘I’m depressed’). She insisted that if only the therapist would provide her with reassurance and answers to her lists of questions, then she would be happy.
In therapy sessions, Serena often struggled to differentiate past from present, losing sight of herself as an adult woman with a high functioning career. Imagery Rescripting became an opportunity to replay her childhood fantasy of being ‘rescued’ rather than emotional processing and facing the reality of the past. This only served to strengthen her belief that if only the therapist could meet all of her needs, and provide all of the answers to her problems, all would be well. Attempts to bring her Healthy Adult self into the images were either passively played out with minimal internalization, or resisted altogether, claiming “I cannot…I need you to do it.” Between therapy sessions, Serena’s behavior became increasingly regressed. Gaps between sessions, such as when the therapist took holidays, were met with aggressive outbursts via email. When the therapist attempted to address this by enquiring about underlying emotions in the following session, she would weep silently, but was unable to verbalize her distress. There was a implicit expectation that the therapist should know what she felt and completely gratify her needs. At the same time she found it difficult to show respect for the needs and boundaries of the therapist.
No matter how hard the therapist tried to meet Serena’s needs, it was never enough. In Helpless Surrenderer mode, Serena consistently expressed irritation and a sense of unfairness at the *limited* aspect of reparenting, while her demands for ever greater care prevented her from taking-in the care that was provided.

The strategies of the Helpless Surrenderer (Careseeker) are consistent with the ‘Obsessive Coercive’ strategies described within the Dynamic Maturational Model of Attachment, i.e., “exaggerated display of feelings to coerce attachment figures into responding… associated with uncertain expectation of threat or danger” [(28), p. 2]. These protest behaviors may include an array of coping responses, including angry demands directed at the other person, distancing and behaving with emotional coldness to punish the other, feigning helplessness, and giving the impression of needing to be rescued from self-inflicted risk, frequently alongside a pattern of idealizing others. Ultimately, the child has learned to exaggerate vulnerability with the underlying intention of seeking help and care, in order to allay underlying authentic feelings of helplessness. These strategies are characteristic of the pull-push strategies of alternately seeking and then denying care, which is characteristic of borderline personality disorder (29). These coping patterns are largely in place by early infancy, becoming entrenched over the person’s lifetime (28).

###### Common countertransference reactions

- At first, the therapist may feel a strong sense of guilt and responsibility to rescue the client from their distress and produce endless new solutions and strategies to alleviate the client’s difficulties. The therapist may be overfunctioning in a Self-Sacrifice/Rescuer mode (23), characterized by rescue fantasies of ‘saving’ the client from their distress. They may also work hard to draw in other clinicians to assist, while feeling frustrated when they do not respond with the same sense of urgency.
- The therapist feels idealized by and/or fused with the client – as if the client finds it difficult to tolerate the thought of them as a separate person. As such, the therapist may be left with a sense of being ‘used’ rather in the same way as an addictive substance, rather than as a relational being.
- Therapists may avoid discussing termination of therapy, in a collusive attempt to avoid re-traumatizing the client. Further, they may collude with avoidance of experiential techniques, believing the client’s claims that they ‘do not work’, or that they are too fragile/unwell/at risk. Instead, the therapist may find themselves being drawn into a version of solution-focused therapy based on strategies and techniques rather than adhering steadfastly to the powerful emotion-focused techniques of Schema Therapy.
- The therapist begins to feel inadequate, overwhelmed, trapped, helpless and stuck when nothing seems to ‘work’ (30). They may sense that they are being ‘manoeuvred’ and that whatever they offer does not seem to be absorbed. Over time, the countertransference can erode the therapist’s empathy and lead to feelings of increased guilt, failure, and ultimately burnout. The therapist may experience a sense of guilt at their dwindling feelings of compassion, and try to ‘push through’, in spite of their intuitive sense that there is something inauthentic about the way that this mode presents.

##### Helpless Surrenderer (Resigned)

The covert version of the Helpless Surrenderer mode takes the form of a ‘Helpless-Resigned’ coping mode. This version more often manifests with clients with avoidant attachment and rigid/overcontrolled difficulties, including Cluster C personality traits. In this mode, the client presents as emotionally and/or physically fragile and helpless. On the surface, the person may manifest other more dominant avoidant or overcompensatory coping modes that appear reticent to engage and may even take pride in being self-sufficient. In this context, the Helpless-Resigned mode frequently manifests in the background, through dissociated symptoms, somatization, physical illness, eating disorders, and medically unexplained pain and other symptoms.

In our clinical experience, the Helpless-Resigned version of this mode commonly develops in the context of a childhood whereby the child’s emotional self (Vulnerable Child) is experienced as ‘invisible’, and they have learned to perceive their needs as an imposition on others. The childhood is characterized by practical caregiving, with minimal emotional warmth, affection, ‘holding’ and attunement. Expression of emotions or needs may be met with guilt-inducing messages, either overt or more subtle, such as facial expressions of disappointment, hurt, or exasperation. Getting needs met becomes linked to feeling guilty, obligated, and a burden. The child has given up hope, and resigned themselves to not getting their emotional needs met. They have learned that retaining hope only leads to disappointment and shame. The Helpless-Resigned mode emerges as a means of translating inner vulnerability into an outer physical symptom or behavior which can be ‘seen’ and understood by others – especially others who have low emotional maturity and only react to that which can be seen with the eye.

When core emotional ingredients have been consistently missing, or have come at too high a cost, the child gives up hope of getting their needs met, while learning to protect themselves with a wall of resigned hopelessness. ‘Not-needing’ and ‘not-hoping’ become the safest option, designed to protect from the underlying pain of despair and loneliness of invisibility. One of the roles of Helpless Resigned mode is therefore to protect from the painful and humiliating experience of hopes being dashed by keeping expectations low, while simultaneously ‘showing’ vulnerability on the ‘outside’ via problems, physical body manifestations and symptoms that keep the underlying emotional vulnerability hidden.

A case example illustrating the Helpless-Resigned mode is provided below:

**Table:** 

Tanya, a 28-year-old woman presented with Anorexia Nervosa and chronic (medically unexplained) throat pain. She had been unable to eat without experiencing intolerable nausea, leading to avoidance of eating and a chronic pattern of weight loss. She worried that her symptoms indicated a serious physical condition. In hospital, Tanya became highly distressed during mealtimes, frustrated that the treatment team did not appear to take her pains seriously. She described feeling misunderstood, dismissed, and not taken seriously.
In therapy sessions, Tanya carefully chose her words as if her concern was that she might burden the therapist with her problems. At the same time she was concerned that she would be misunderstood if she did not explain her difficulties in the ‘right’ way. Her main focus was on describing her physical pain, her fear of what this might mean in terms of serious illness, and her frustration that nobody seemed to understand how difficult it was to manage this. In spite of her underlying distress, she described her pain and struggles in a detached manner, frozen and resigned to her predicament, like a ‘deer in the headlights’.
Assessment of Tanya’s past revealed that she had been parentified since early childhood. Her mother had severe health problems and spent a lot of time in bed, while her father was away most of the time with work. From the age of 5, Tanya was increasingly expected to cook meals, clean the house and take care of her young sibling, while nursing her mother. Tanya was lonely, isolated, and struggled to relate to the other children at school. When other adults (aunts, uncles) were present, their focus was on her mother’s illness, and she largely remained in the background. Tanya felt unable to express her emotional suffering or ask for support, as it always seemed that her mothers’ needs were so much more legitimate and deserving of care. On the rare occasions that she did mention her own difficulties, she immediately felt guilty and an imposition. She learned to filter her thoughts before speaking in an attempt to avoid being seen as a burden.
Over the course of therapy, Tanya began to trust her therapist and explore childhood experiences of neglect, loneliness and despair. Gentle exploration of childhood enabled her to understand that she had implicitly learned that only physical symptoms would be recognized as legitimate and worthy of care within her family of origin. On the one hand her Helpless-Resigned mode had protected her from the pain of continual disappointment, while on the other it had enabled her to translate her emotional needs into a ‘language’ that her parents would understand, respond to and view as deserving of care.

This manifestation of Helpless-Resigned mode is consistent with early writings based on health-related anxieties and preoccupations, in reference to the process referred to as *secondary gain* or embracing a *sick role*. Although the concept of secondary gain was introduced by Freud, it has since been further elaborated to refer to presentations in which manifestations of physical pain and frailty may function as displacement of inner emotional loss and neglect. In particular, Engel (31) and Van Egmond (32) describe instances [of] origins whereby the child has experienced cold or distant parenting except in instances when they have been physically ill or in pain. Over time, this response is intermittently reinforced as a legitimate means of eliciting care (33). The case of Tanya provides an example of a client who has learned within the context of her family of origin to both understand and communicate her emotional needs through the outward appearance of her body. Her need for care was expressed through manifestation of physical illness – the only way she had learned that parents would view as legitimate of care. The way this can be learned in childhood, and the secondary gain that reinforces it, is a widely recognized clinical phenomenon, which functions as overcompensation for early emotional deprivation.

Although Helpless-Resigned mode can manifest in a similar way to the Complaining Protector (Help-seeking-help-rejecting), the key distinction between the two is that the former tends to present more passively, giving the impression of having given up on care. In contrast to the Complaining Protector, the motivation of the Helpless Surrenderer is not to create distance from the emotions altogether, but rather to give in to the feeling of suffering, albeit in the form of global diffuse suffering rather than the authentic distress of the Vulnerable Child mode. It has a flavor of surrendering to ‘misery’ (like a deflated balloon), such that the person insists that others are not understanding or helping them enough, or in the ‘right’ way. It is this secondary hopelessness and resignation that functions as a protective shield from core experiences of hopelessness and aloneness. There is a sense that the person is somewhat unreachable, and often highly ambivalent due to the costs of being helped by others.

###### Common counter-transference reactions

- Empathy, compassion, sense of responsibility to generate solutions to ‘solve’ the client’s difficulties through concrete, tangible advice and strategies.
- Frustration, irritation and overwhelm when these strategies appear to have little or no impact.
- Avoidance of discussing and working toward an ending in therapy, in spite of lack of progress.
- Feelings of inadequacy, incompetence, helplessness when therapeutic interventions appear to have minimal impact, followed by an urge to refer on to a more ‘competent’ therapist (30).

### Avoidant pseudo vulnerable modes

#### Complaining Protector mode

The Complaining Protector includes two versions, both involving a presentation of ‘pseudo vulnerability’. The Help-seeking-help-rejecting version is more common in clients with chronic pain and cluster C disorders. The Belligerent version is more common in clients with cluster B traits, addiction, or clients in in-patient clinics and forensic settings.

##### Complaining Protector: Help-seeking-help-rejecting

**Table:** 

Anna, a 32-year-old woman, starts the session by describing in great detail that she has not been feeling well this week. She has suffered from numerous stomach problems and sought help from the doctor, but the doctor did not give her the ‘right’ medication. In general, she feels that doctors do not take her seriously. She has conducted her own ‘research’ and discovered that there are much better treatments available for treating her digestive problems. She feels frustrated that she had to find these herself instead of the doctor. She continues by explaining that because of the ‘wrong’ medication the doctor gave her, she has also had difficulty sleeping, and that has also affected her mood. The therapist offers to take a close look at the situation with the doctor, and her feelings regarding it. Anna says it’s pointless to discuss this, as she is not planning to see this doctor anymore.

The Complaining Protector is an avoidant coping mode, which functions to keep a distance from vulnerable emotions, and avoid genuine connection with others (11). It creates a ‘smoke screen’ of complaints, and invites people to listen, but under the assumption that these problems will not be solved. In other words, the client who is in this mode is not looking for genuine care from the therapist, and rejects any attempts to offer solutions. If given the time and space, this mode can easily take over the session, seeking a ‘listening ear’ and maybe some sympathy from the therapist.

The description of this mode originated from the literature based on chronic pain, whereby complaints of physical pain function as a means of diverting the person’s attention away from emotional loss and neglect while simultaneously drawing attention to their suffering to show others that they are not doing enough to help (11). This ‘sick role’ phenomenon as described by Barsky and Klerman (34) has been linked to a range of medical presentations.

The emotional presentation of this version is mostly ‘flat’, but sometimes it may involve slight annoyance. The mode often covers strong emotional deprivation (e.g., “no one will take care of me”) or themes of mistrust (e.g., “If I show people my pain, they may use it against me”). When clients have learned that people will not truly care for them or soothe their pain, they may put up a ‘wall’ to protect themselves from being disappointed all over again. This ‘wall’ conveys the message of suffering, but does not let anyone in to connect with the person’s authentic vulnerability. In doing so, this also signals to the therapist: “I will tell you all about my problems and how people do not take care of me, but I will not let you take care of me either”.

The ‘sympathy’, or the ‘listening ear’ that the therapist often provides to this mode only creates more distance from the true vulnerability of the client. It makes the mode ‘bigger’, and only reinforces the avoidant coping. Further, offering concrete ‘solutions’ will not help, as the mode then rejects these (“I already tried that, it did not work,” or “That will not work for me”). In order to bypass this mode the therapist will mostly need to confront it in an empathic way, referring to it’s protective function for the client, as well as the effect it has on the therapeutic relationship (for a detailed example of empathic confrontation see Discussion). Once the client can gain a sense of distance from this mode, they may become more open to talking about their true disappointments within core relationships, (e.g., mainly caregivers), their underlying loneliness, or even to share more of their deep frustration and anger. Only then, can the process of healing begin.

###### Common counter-transference reactions

- Feeling emotionally ‘numb’ and trying to think ‘rationally’ about possible solutions.
- Feeling a sense of pressure to come up with the ‘right’ solution to the problem.
- Feeling frustrated or a sense of failure.

##### Belligerent Complaining Protector

**Table:** 

Rita, a 42-year-old woman, is unhappy in her marriage. She often complains about her husband, provides many details about how he mistreats her, and tells the therapist about all that he does wrong. “*I do not understand why he keeps acting like that!*” She says. “*Why does he always have to be such a ‘cold’ and ‘careless’ person?*” When the therapist is trying to validate her feelings of frustration, Rita often becomes increasingly agitated. “*Of course I’m frustrated! Do you know what else he did this week? You will not believe it*!” She continues on at great length, elaborating on other situations where he has mistreated her. The therapist asks Rita if she is angry. *“I’m just frustrated,”* she replies. *“Why cannot he just change his behaviors? Can you give me some ideas of how to help him change?”*

This version of the Complaining Protector also involves a core behavioral pattern of complaining, with the motivation of avoiding closeness. However, in this case, the main focus is on *others’ wrongdoings* and it involves a more overt expression of frustration than the Help-seeking-help-rejecting version. The purpose of this mode is to find more and more reasons to complain about how others, for example, are not doing their job, not behaving appropriately, or are mistreating the person. The complaints are directed outwards in a way that leaves the person feeling like a victim. When this mode is active, the person is not capable of self-reflection, or examining the impact of their own actions in the situation. There is a hyper-focus on external detail, while others’ behaviors are perceived as the main cause of the person’s suffering. It is often associated with a tendency to externalize problems (and externalization of shame), and is more common with clients with cluster B traits.

While the presentation of this mode often involves more emotion than the Help-seeking-help-rejecting Complaining Protector, the anger or frustration should not be confused with the Angry Child mode. The anger of the Angry Child tends to diffuse when emotional needs are met (e.g., such as listening and validation). However, the frustration of the Belligerent Complaining Protector tends to persist and even escalate when given more space. Sometimes it may shift into another pseudo vulnerable mode, with a presentation of either helplessness or a sense of victimization and self-pity.

We identify the Belligerent Complaining Protector as an avoidant mode in that it meets all elements of avoidant coping: e.g. affect, cognitions, behaviors, and desires (16). The motivation/desire is to use complaints in order to avoid closeness or genuine emotions (11). The cognitive aspects involve focusing on others’ wrongdoings rather than on oneself and a tendency toward externalization. The emotional presentation involves mild frustration rather than a full expression of anger or pain. If the complaining behavior is associated, for example, with a motivation to feel powerful/superior rather than powerless/inferior, and when the flavor of that mode is no longer pseudo vulnerability but rather aggressive or demeaning – then clearly this is no longer a Belligerent Complaining Protector but rather an overcompensatory mode (such as Self Aggrandizer or a Bully and Attack mode).

The Belligerent Complaining Protector is relatively easier to identify in in-patient clinics and forensic institutes, as it becomes ‘infectious’. Clients may unconsciously collaborate with this kind of avoidance and spend therapeutic sessions talking about how the team is not doing its job well enough, how meals are not satisfying, and how the rules do not make sense. The focus is primarily on others’ behaviors. Group sessions can easily be taken over by this mode, emerging into an hour and a half of complaints.

However, in private practice, the mode is much harder to recognize and is often very misleading, especially when complaints are based on ‘reality’. In other words, the therapist may often feel that there is some ‘truth’ to the complaints, and be more prone to collaborate with this mode and perceive the client as a ‘victim of circumstances’. However, the fact that complaints can be justified by real events is not evidence of the client’s emotional state in the session. The main questions that should be asked are: *Which side of the client is telling the therapist about the distressing reality? What is the function of this side*?

For example, in Rita’s case, despite the fact that her husband mistreats her, the complaining mode stands in the way of therapeutic progress. It focuses only on her husband’s misdemeanors, blocking self-reflection and mentalization, thereby preventing the therapist from reaching her underlying vulnerability. If she dared to look inside and lower the ‘wall of complaints’, she might have to feel emotions that are very painful and uncomfortable. For example, she may be faced with her deep fears of separation, which influence her ability to leave her husband. She might be faced with her genuine pain as a child who grew up with an abusive father. If the mode was not there to shift the spotlight, the therapist might have seen more of her shame, and her deep belief that she is not worthy of being with anyone who truly loves her.

The main intervention with this mode involves empathic confrontation. However, this mode is more sensitive to being confronted. With cluster B clients, therapists may often experience some of the anger directed toward them. As the client’s ability to reflect under the influence of the mode is relatively low, confrontation with the mode may lead to a momentary perception of the therapist as the ‘depriving’ and the ‘abusing’ caregiver (i.e., cast as the Perpetrator on the Drama Triangle). The client may also shift toward another pseudo vulnerable mode, for example, the Attention/Recognition Seeking ‘Do not blame me’ mode. Schema Therapy always involves working with modes in the ‘here and now’ and following the modes as guidance for interventions.

### Overcompensatory pseudo vulnerable modes

#### Attention/recognition seeking mode: ‘Do not blame me!’ version

The Attention/Recognition Seeking mode involves over-compensatory coping, and tends to display emotions in a highly superficial or rather dramatic way. It operates by increasing the ‘volume’ of some emotions, until they are so ‘loud’ that you cannot hear the genuine emotions underneath. Indeed, when this mode presents with ‘vulnerability’, it often feels sudden and dramatic, or even overwhelming to the observer. In many cases, the function of the mode is to gain attention or acknowledgment, but here the presentation of vulnerability also serves as a tool for ‘neutralizing’ others and escaping responsibility or blame.

**Table:** 

Richard (55) and Lia (47) are in couples therapy. Currently, Richard is scared and worried about their financial situation. He is working three jobs and feels a great sense of burden. Lia stopped working a year ago (she resigned from her job). Richard has tried several times to have a serious conversation with her about the financial situation, but every time he gets the same reaction. Lia immediately starts crying*. “I am really suffering and depressed, do not you see that?? Why are you bringing up this topic when you see I’m not well? I wish I could tell you how hard life is for me these days.”* She cries even more and he hugs her. He has given up on trying.

Another example:

**Table:** 

Sarah is a 25-year-old patient in an in-patient clinic. She has a severe history of early childhood trauma. During the weekend on the ward, she was involved in some boundary-crossing behaviors with another client. Her therapist wanted to address this in their session.
Therapist: “*Sarah, I want to talk to you about what happened last week in the clinic. I heard that you pushed another client, I was wondering how you are and what was going on?”* Sarah bursts into tears. “*I feel so guilty, I’m so ashamed of myself. I was so down this week and felt overwhelmed about what happened with my father. I never told you about it. He hurt me so much.*” Sarah cannot stop crying for almost 20 min.

This version of the Attention/Recognition Seeker is often confused with a Vulnerable Child mode as the presentation of pain can be rather intense, and the ‘themes’ of suffering can be rather similar to the themes of genuine vulnerability. For example, both Lia and Sarah have moments when they can show and share their genuine pain with others. However, in those moments when they are being confronted, their Attention Seeker mode has a different function: it draws attention to their suffering, claiming the ‘Victim position’, and by doing so it gives a clear message: “I am in so much pain that I cannot be confronted or held accountable for my actions,” Or: ‘I am suffering now! Do not confront me!”’ This mode is therefore highly effective in ‘neutralizing’ others.

Moreover, one of the ways this mode operates is through inducing guilt. It signals to others that their attempts to confront have caused the person to suffer. ‘Walking on eggshells’, therefore, is a very common reaction to this mode, along with the intense feeling of shame and guilt, or anger. Furthermore, when this mode appears in the session as a response to empathic confrontation, the guilt-inducing mechanism may also be an indication of the client’s anger toward the therapist. For example:

**Table:** 

John, a 24-year-old client, talks with his therapist about his recent first date. He cannot understand why the woman was not interested in seeing him again. As he describes the events it becomes very obvious to the therapist that John was highly demeaning toward the woman (e.g., Self-Aggrandizer mode). He offers to take a look at this side of John and understand it better. As a response, John looks down…*“Now even you are telling me that I’m defective. My parents were right, I will never find anyone.”*

In his example, John’s expression of sudden ‘vulnerability’ was also inducing a reaction in his therapist, as he felt like he was ‘punched in the stomach’. That is often the case when this mode appears, as the client is expressing his anger indirectly via claiming the ‘Victim’ position. It gives the therapist the clear message: ‘look what you did to me’! By doing so, it ‘punches back’ with tears instead of fists.

The origins of this mode can vary. In some cases, it started as an adaptive way of coping, as the child learned that a presentation of vulnerability could protect them from criticism and pain (e.g., “If I am suffering and crying, no one will hurt me/“If I show my pain now people will stop hitting me”). The ‘Victim’ presentation, therefore, serves as a pre-emptive attack on oneself. In other cases, this mode started as an effective way for the child to get away with things he did not want to do, or with facing consequences for his actions. In those cases, it usually involves an underlying sense of entitlement (e.g., “If I am suffering I can get away with not taking responsibility”).

As with other modes, manifestation differences are seen in different types of personality disorders. For example, clients with borderline or histrionic traits often show a more sudden, extreme, and externalizing presentation of this mode. The behavioral presentation therefore is very loud, and often involves crying. The narcissistic presentation may come across as more covert, guilt-inducing, and it often feels like the client is playing the ‘poor me card’ to get away with their behaviors.

##### Common counter-transference reactions

- Shock, feeling ‘trapped’, anger.
- Guilt and shame.
- Feeling the urge to ‘walk on egg-shells’, trying be very careful not to hurt the client.

#### Self aggrandiser mode: Self-Pity/Victim version

**Table:** 

Paul, a 35-year-old businessman, enters the session looking very tired. “*You will not believe what a week I’ve had*,” he moans. “*Every day after work I had to drive my son to his basketball practice. He did not want to take the bus because it was raining and he was a bit tired. He is 17 you know, it’s a tough age. On the weekend he wanted my help with his homework assignment because he forgot the due date was this Sunday. I had to cancel the plans I’d made with my wife and her family so I could help him. My wife got so mad at me and we had a big fight. She does not get it. No one gets it. I give so much and then she dares to get angry at me? He is her son, too!*” The therapist then tries to explore Paul’s tendencies to sacrifice his time (and also his relationship) for the sake of making his son happy. Paul is not happy with the therapist’s comments. “*I have no choice. Everybody is telling me the same thing*.”

This ‘narcissistically abused’ version of a Self-Aggrandizer mode can present suffering in a rather unique way (12, 35–39). In this version of Self Aggrandizer, it is the suffering itself that gives the person a specialness or exceptionality, and therefore it cannot be relinquished. They may show up as ‘virtuous victims’, insisting that their suffering and pain is greater than that of others (12). In the example above, Paul does not reveal his genuine emotions to the therapist (for example, his sadness, loneliness, or anger), but rather presents with a theme of ‘poor me’. Indeed, the narrative of the martyr version is often related to the underlying message: ‘Poor me, I am suffering because I do so much for others, but I have no choice’.

In contrast to the genuine Vulnerable Child mode, the Self-Pity/Victim is not looking for soothing, closeness, or reassurance from the therapist. It rather looks for pity, mirroring, or admiration. The client’s perception of himself as a ‘martyr’ or a ‘saviour’ often provides a sense of self-importance, meaning, or control, and therefore they are not willing to give up on that version of reality. The assumption that others are either helpless, incompetent, or completely dependent on him may also serve the grandiose perception of himself. The presentation of pseudo vulnerability is usually not accompanied by crying, but rather by moaning, showing tiredness and exhaustion, or rolling eyes (often, a classic sign of a Self-Pity/Victim).

Possible interventions with this mode include exploring the positive and beneficial aspects of the client’s tendency to self–sacrifice. The therapist could also refer to the effect of this mode in the therapy relationship, using self-disclosure. Exploring genuine vulnerability could start by referring to the client’s experience that no one understands him, and how it makes him feel. Only when the client is ready to explore how their life or their self-esteem might look if they stop acting like a ‘martyr’, can they move forward to process the pain and grief involved in giving up this coping mode.

## Discussion

### Differentiating pseudo vulnerable from authentic vulnerable modes

Signs that can be used by therapists to differentiate pseudo- from the authentic vulnerability include the following key points described below and in Table 2.

**Table 2:** Distinguishing Pseudo Vulnerable modes from authentic Vulnerable Child should be in capitals throughout article.

Vulnerable child	Pseudo vulnerable modes
Emotions are experienced and expressed authentically – “*I am sad, lonely*”	*Talks about* emotions, often using ‘pathology’ focused language, but without nuance of true emotional felt-sense – “*I am….depressed, suicidal…*”
Goal is to connect	Goal is to control and avoid. Connection must be ‘earned’ through describing or showing one is sick or helpless.
Emotion feels authentic	Emotion feels slightly staged, histrionic, or out of sync with situation.
Attachment feels authentic (core feelings of abandonment, fear, grief)	Attachments have addictive quality. Interpersonal connection is viewed as opportunity for sympathy, care, strategies, advice, practical help or ‘fixes’ rather than as a source of intimacy.
Others feel empathically connected	Others feel urge to rescue and/or trapped/‘used’/burned out.
Elicits empathy from others	Elicits Pity/Sympathy/Rescue (and reassurance, advice, solutions) via helplessness, hopelessness, guilt-inducing, sickness, obligation, complaining, ‘wounded-ness’, risky behaviors (or threat of).
Can internalize Healthy Adult messages (and allow own Healthy Adult to take on self-parenting role)	Refuses care from own Healthy Adult. Pays lip service to therapy but perceives it as an opportunity to be ‘fixed’ rather than a collaborative process.
Feels abandoned, fear, grief, and (secondary) shame/guilt regarding unmet needs	Protects from shame or guilt that would be elicited by direct expression of core needs for love, connection by finding passive or ‘undercover’ ways of expressing emotions and needs.

#### Talking-about vs. being-in vulnerability

In genuine Vulnerable Child modes, emotions such as sadness, fear, shame, or guilt are felt deeply, and described in a relatively nuanced manner. Moreover, because they involve genuine emotions, their links to specific schema meanings, and underlying core unmet needs, are evident (e.g., “I’m feeling scared and sad because my partner left me…”). In contrast, pseudo vulnerability has a more abstract or global and undifferentiated, flavor. The person talks about emotions, or about their diagnoses, in a distant, abstract, intellectual, or overly rational way, or in a vague way that lacks detail and elaboration (e.g., “I am depressed, suicidal.”). Clients may talk at length about their diagnoses as evidence of their suffering, while evading any discussion about distress that directly links to underlying unmet needs. Further, in the case of the Self-Pity/Victim(Martyr) mode, suffering is worn almost as a ‘badge of honour’, rather than involving genuine feelings of sadness or distress.

#### Therapist is viewed as a ‘fix’ rather than a source of connection

Another important signal that a pseudo vulnerable mode is manifesting is that the attachment takes on an addictive quality, whereby other people become a source of pseudo-connection (reassurance, advice, sympathy), rather than authentic connection. As the connection is based on the therapist providing sympathy, reassurance and short-term relief or solutions, contact provides temporary relief, but with no lasting effect. With no internalized healthy capacity to self-regulate, the therapist thereby becomes the ‘fix’ that the client craves to bring relief to their distress and suffering.

#### Dependent child mode vs. Helpless Surrenderer (the ‘Careseeker’ version)

Both a Dependent Child mode and a Helpless Surrenderer coping mode may present with feelings of helplessness. However, the Dependent Child mode seeks soothing and reassurance in a direct manner, due to feelings of fear and overwhelm with adult life 36. The client therefore may present as regressed, with emotions and behaviors indicating a sense of panic, fear, and difficulties with regulating themselves. They may search for an adult to complete tasks for them. The Helpless Surrenderer, however, does not reveal its genuine fear of separation or independence. Instead, it tries to induce guilt by convincing the therapist/caregiver that he is, in fact, cannot be left alone or function by himself. The client therefore may insist that he is ‘too fragile’ ‘too sick’, ‘too suicidal’, or ‘too depressed’. Dependent Child mode is often a result of authoritarian or overprotective childhood, whereas a common background for clients with Helpless Surrenderer is a lack of emotional availability, dismissive style of caregiving, often alongside high standards/expectations for achievement. When attachment needs are provided, such as soothing and care, and autonomy needs are encouraged, a Dependent Child will eventually be able to grow and separate. However, providing those ingredients of care to a Helpless Surrender will most likely reinforce the secondary gain of the mode, and the unmet needs of the client will remain under the surface.

#### “Do not confront me!”

In overcompensatory modes such as the Attention/Recognition Seeker, pseudo vulnerability can emerge as a warning to the therapist: “I am really struggling and fragile today, so do not confront me!”/“I am in so much pain that I am not accountable for my actions.” The resulting countertransferential reactions that arise in the therapist include feelings of guilt, ‘walking on egg-shells’, anger, feeling ‘punched in the stomach’, or ‘handcuffed’ by the client. In contrast, genuine vulnerability usually evokes feelings of empathy and compassion in therapists.

#### Reactions to confrontation

Another sign of pseudo vulnerability is indicated by the way the client responds to being confronted by the therapist. When the therapist invites the client to look at his or her own behavior, the client may flip into another maladaptive coping mode. For example, the client may keep the therapist at a hostile distance (Angry Protector mode), sending the message, “Back off!,” or accuse the therapist of mistreating, abusing, or rejecting her (Bully and Attack mode). It is also very common that a confrontation with a pseudo vulnerable coping mode (for example, with a Helpless Surrenderer) will result in another presentation of pseudo vulnerability (such as Attention/Recognition seeking ‘Do not blame me’). These maneuvers have the effect of undermining the therapist’s efficacy, leaving them feeling preoccupied by their own Inner Critic. They then may abandon any attempt to persist with the therapeutic intervention. This dynamic is evidence that the therapist is dealing with an army of coping modes rather than a genuine child mode.

#### Avoiding emotions

A further indication is how the client responds when the therapist attempts to explore emotions. If the therapist attempts to explore feelings of anger (for example, when the client is in a Complaining Protector), the client may admit to mild feelings of frustration or irritation, and then quickly reverts back to complaints. If the therapist empathizes with and validates the client’s sadness, fear, or distress, the client usually will not experience relief, but rather they will complain more, or flip into another pseudo vulnerable coping mode.

#### Avoiding Imagery Rescripting techniques

Finally, in comparison with clients experiencing genuine vulnerability, those in pseudo vulnerable modes are less willing to try Imagery Rescripting techniques, due to their capacity to evoke painful emotions. Instead, they insist on focusing on discussing current global situations in their life and/or the problems they attribute to the other person in an abstract, distant way.

### Steps for working with pseudo vulnerable modes

Empathic confrontation and limit setting are key techniques for working with pseudo vulnerable coping modes. In practice, this can be difficult to implement as pseudo vulnerability is part of the armory that enables them to avoid facing the pain and sadness of past neglect and losses in their lives. The therapist must gently but firmly confront the client with the past and present realities of disconnection, loss and neglect that they are avoiding and help them to process the grief and anger that emerge when this reality is faced. When pseudo vulnerable modes are present, the reparenting ‘recipe’ must involve frustrating the client sufficiently to enable them to face what they are trying to avoid. While extensive guidance on clinical interventions for working with coping modes are available within other resources [e.g., (14, 36–41, 45)] some key steps for working with these modes are described in the following section.

#### Explore origins of the mode

The therapist’s first task is to help the client to recognize that this mode emerged in childhood as a form of coping with being ‘unseen’ and ‘unheard’. Whenever the child expressed emotions and needs directly, this was usually discouraged in the family environment. This conversation might begin through exploring the messages that the client learned in childhood which originally elicited feelings of guilt and shame in regard to expressing vulnerability and emotional needs. From there, the therapist can explore ways in which the coping mode enabled them to manage their childhood circumstances and unmet needs. The therapist can also explore ways in which these modes may have developed within past generations as a result of oppressive circumstances and cultural biases, and been passed on through the generations. Discussing the pseudo vulnerable modes can elicit strong feelings of shame and/or guilt. Therefore, the therapist must gently but persistently introduce the mode to the client’s conscious awareness, as well as exploring possible secondary gain both in the past and present which may drive ambivalence about acknowledging and potentially relinquishing this mode.

#### Psychoeducation regarding protective functions of the mode

A useful metaphor for understanding the protective functions of the mode is Matryoshka dolls, in which each of the increasingly smaller versions of the same doll are found within it. The therapist assigns each of the client’s coping modes to one of the layers of the doll. Each layer of the doll protects the other, smaller layers. The therapist explains that the pseudo vulnerable mode is the second to smallest, while the Vulnerable Child is the smallest one, barely visible. The therapist explains that reaching the Vulnerable Child in therapy will enable them to learn healthy ways of getting needs met through authentic connection.

The Drama Triangle (24) can be a useful tool for psychoeducation and exploring the ways in which pseudo vulnerability operates within the specific dynamics of the client’s family. Introducing the concept of pseudo vulnerable modes as one component of the overarching drama triangle ‘system’, can have a significant de-shaming effect as the client recognizes that this is simply a role that they have learned within the context of their family dynamics. This opens the door to an exploration of the different roles that other family members hold (e.g., Rescuer, Persecutor, Victim), how these are played out, and the fears that each person has of ‘stepping off’ the triangle. The therapist can also guide the client to explore the ways in which being in the ‘Victim’ position may in fact be meeting a need in other family members. By exploring ways in which the pseudo vulnerable modes play an implicit role within their own family, the client can begin to free themselves from the blame and shame or having adopted pseudo vulnerability as a means of coping. The Drama Triangle may also be helpful in providing a framework for exploring with the client the possible dynamics in the therapy relationship.

#### Explore pros and cons of the mode

Exploring the pros and cons of the mode can help the client to identify both advantages and disadvantages of this way of coping. Maladaptive coping modes have advantages (e.g., protection from pain, secondary gain through attention), as well as disadvantages. Helping the client to weigh the benefits and costs of the modes is necessary preparation for beginning to change them. Using Chairwork dialogues (10) can facilitate the process of exploring pros and cons. The therapist invites the client to move to a different chair and play the role of his or her pseudo vulnerable mode. He interviews and explores the fears and intentions behind the modes and explores healthier alternatives.

#### Limited reparenting

Limited reparenting requires a responsive balance of both nurturing ingredients such as warmth, and attunement and care, and boundary-based ingredients including limit setting, and empathic confrontation. This includes an element of ‘imperfect care’ whereby care is balanced with healthy levels of frustration. Therapists must be aware of their own Self-Sacrifice/Rescuer modes that can be drawn into rescue dynamics, blocking opportunities for the client to learn healthy skills for self-regulation when needs are not met 100% of the time. ‘Good enough’ care, when provided in a developmentally appropriate way, provides opportunities for the client to learn patience, tolerance, respect for others’ boundaries, and resilience (42, 43). However, healthy frustration is a key ingredient that needs to be sufficiently balanced with empathy and care in order to provide the impetus for clients to relinquish unhealthy coping patterns, and face the difficult and often painful process of emotional processing. An important difference between reparenting from a Healthy Adult (as opposed to a Rescuer mode stance) is that the therapist is actively working toward encouraging the client’s own Healthy Adult side to take an active role in learning to reparent their own Vulnerable Child. The Healthy therapist’s role is to facilitate that process through coaching and reinforcing the client’s efforts to take some ownership of their own recovery.

The therapist should persistently help the client to recognize when the pseudo vulnerable modes appear both in and out of sessions, while making it clear that progress in therapy depends on connecting with the Vulnerable Child mode. By validating the role of the pseudo vulnerable mode in childhood and helping the client to differentiate past experiences where it wasn’t safe to express authentic vulnerability from the present therapeutic relationship, the client can begin to feel sufficiently safe to begin to experiment with expressing authentic vulnerability. Indeed, a key dimension of reparenting, especially when dealing with pseudo vulnerable modes such as Helpless Surrenderer and Complaining Protector, is facilitating autonomous functioning and the client’s belief in himself. This requires the therapist to have a realistic estimate of the client’s capacities even when the client does not, helping him to develop these capacities and test out unrealistic fears about his vulnerability or sources of pain. It can be counterproductive to launch into experiential work when these modes continue to dominate. In our experience, although clients in these modes may pay lip service to Imagery Rescripting, the impact will be limited and short-lived, with little if any emotional processing having taken place. The limited reparenting messages will therefore provide short-term reassurance, reinforcing the therapist’s role as the Rescuer, rather than leading to a genuine connection that is internalized. When the therapist does introduce exercises such as Imagery Rescripting and Chairwork, they should check-in regularly on the authenticity of the client’s emotions to ensure that they are accessing the Vulnerable Child mode. Further, the client’s own Healthy Adult mode can be brought into these exercises early on, even simply in an observer role until they are coached to gently take on some of the reparenting their own Vulnerable Child.

#### Empathic confrontation of pseudo vulnerable modes

Empathic confrontation is the most important intervention for working with pseudo vulnerable coping modes in order to reach the authentic vulnerability that lies beneath. Pseudo vulnerable modes can be quite persistent in the face of the therapist’s attempts to confront them. Therapists must be determined to stick to their methods. The therapist confronts the client’s modes in real time, “holding a mirror” to them so that the client can see them, too, and begin to understand the reasons behind them. In some cases new modes may arise, which requires sequential empathic confrontation (23). For example, after confronting the Helpless Surrenderer mode, the Self-Pity/Victim mode may appear with the message “How could you confront me when I’m suffering so much!” Empathic confrontation helps the client to recognize that while their pseudo vulnerable modes helped them to avoid emotional pain in the past, they now block authentic connection. Because they prevent others from empathizing with them, they reinforce feelings of being misunderstood within current relationships. The therapist draws attention to the deleterious effects of this coping mode on the therapeutic relationship, and then expands this perspective to help the client to see how it relates to their relationships with others and to the wider issues that originally led them to attend therapy. In our clinical experience, therapists often feel inhibited from confronting pseudo vulnerable modes, especially the Helpless Surrenderer. However, empathic confrontation with a coping mode provides true validation to the Vulnerable Child, and is necessary for the client to make progress. It emphasizes the therapist’s willingness not to give in to a ‘pseudo relationship’, but instead insisting on creating a genuine connection where true reparenting can take place.

The steps involved in empathically confronting pseudo vulnerable modes are summarized below.

### Empathic confrontation with pseudo vulnerable modes

**Table:** 

1. Therapist points out coping mode is trying to convey an important message: “I know that there is a side of you that is here just now, trying to tell me something important….but the message is getting a bit lost in translation. I really want to understand…” 2. Highlight origins and function: “As a child you learned that…your emotions and needs were too much. You grew up feeling that your needs were wrong, or a burden on others. And this side of you picked up the mantle and tried to find a way for you to be ‘seen’ and heard in your family. That side was so clever by trying to help you to get what you needed as a child.” 3. Highlight that this mode blocks connection: “Your needs are normal. Every child needs to connect/be taken care of/ to feel loved/ to be seen and noticed. Your needs were never a burden, never too much. When I see glimpses of that vulnerable side of you underneath, I want to connect. That side of you missed out in the past, and I do not want her to miss out again in the here and now. She deserves my care, and you do not have to prove that to me. I’m already here. I see how loveable and deserving you are.” 4. Self-disclosure to highlight effects on wider presenting problems:… “There’s a side of me that really understands why you are working so hard to be seen… But there’s another side of me that feels frustrated by not being able to reach you and get closer to the vulnerable side of you that’s underneath. When you try to control people in this way, that vulnerable part cannot get the care she really needs…This makes me worry that others might react this way too….and that they won’t take you seriously. Then your vulnerable side misses out again, and you end up feeling more lonely, and depressed.” 5.Check in: “How are you feeling now? I wonder what you heard just now when I brought this stuff up? I know that sometimes your Inner Critic gets in the way at times like this …” 6. Reparent: “I really think that others will want to connect with you if they see the real you underneath. I see this sweet and deserving side of you that is easy to care about. I know this side of you has suffered so much, and I want to hear from this side of you that is longing to be seen and cared for…” 7. Behavioral focus: “Maybe we can do some work together on looking at how you can tell when it’s safe to show your vulnerable side to others, and when it’s not? And we can work together on finding ways for your Healthy Side to stay more connected to that vulnerable side inside of you that sometimes gets lost underneath all of the parts that are trying to protect you…what do you think?”

### Limit setting with the mode

Even after repeated empathic confrontation of the pseudo vulnerable modes, clients may still not be ready to give them up. These modes represent the client’s comfort zone, albeit one that blocks genuine emotional connection. For this reason, therapists may need to set limits on the modes, which otherwise will continue to take the Victim role, present an endless litany of complaints, or insist the therapist provide a quick-fix solution. Under these circumstances, the therapist may need to interrupt the client, stopping the coping behavior that is taking over the session. The therapist needs to be compassionate, but also firm, acknowledging the function that the mode serves, while at the same time, making it clear that it is not in the client’s, nor the therapist’s, interest to allow the mode to dominate the session. By preventing the pseudo vulnerable mode from dominating the session, the therapy is able to move forward.

### Reaching genuine vulnerability

Pseudo vulnerable coping modes require persistent efforts to reach the genuine vulnerability that lies beneath them. Over time, repeatedly confronting and setting limits on the client’s modes opens the gateway to authentic emotions (e.g., sadness, fear, shame, anger), associated schema meanings (e.g., “I’m worthless.”), and unmet needs (“I need to feel seen, loved.”). When the genuine Vulnerable Child mode emerges, the therapist’s limited reparenting can finally provide the healing that the client so desperately needs. The client is able to experience painful feelings and grieve for what has been lost. He or she develops greater self-nurturance, acceptance, and personal agency (18), the signs of an emerging Healthy Adult mode (10).

### Implications and future directions

The pseudo vulnerable modes described in this paper build on those described in previous papers, such as Complaining Protector, Attention-Seeker, Self-Pity/Victim, and additionally describe two versions of the new Helpless Surrenderer mode: Helpless Surrenderer (Careseeker) and Helpless Surrenderer (Resigned). In our clinical experience, each of these appear to manifest differently. However, we recognize that it can often be difficult to cleanly separate avoidance, surrender, and overcompensatory aspects of the pseudo vulnerable modes, such that certain modes may reside at the overlap between these categories. Differences between [pseudo vulnerable] modes may to some extent be driven by such factors as temperament as well as specific early attachment experiences (23, 44).

Fundamental questions remain regarding the nature of modes, including anomalies across the literature regarding different formulations of the same mode, involving different putative forms of coping. For example, whereas Arntz et al. (44) conceptualize ‘resignation’ as a manifestation of Child Modes, our perspective aligns with that of Edwards (23), whereby resignation represents an attempt to cope by blocking the activation of the schema and/or to control the outcome. Whereas Arntz et al. (44) conceptualizes overt careseeking and clinging behavior as an externalizing child mode, we would argue that this has a function to seek or maintain attachment – and therefore is more accurately conceptualized as a coping mode (Helpless Surrenderer – Careseeker). These differences in conceptualization suggest the need for further research to resolve these points of disagreement. Further, the notion that specific modes may actually represent blends or mixtures of other states, such as proposed by Edwards (23), needs to be further clarified to move the field forward, particularly as new mode descriptions continue to be added to the literature.

At the same time, introducing new mode descriptions also raises important questions about the nature and classification of modes. First, with the increasing number of modes that have been proposed by various authors [see (23)], what is the proper balance between comprehensiveness and parsimony? Young et al. (10) introduced the mode concept as a heuristic model for clinical practice -- a practical approach to making decisions when faced with extreme emotional states. The 10 modes that they described were not intended to be a complete theory, nor even a comprehensive description of the range of possible modes. In fact, they deliberately limited the number of modes to 10 to make the model simpler and easier to use (10). In contrast, in a recent review, Edwards (23) noted that there are now over 80 modes described in the clinical literature on Schema Therapy. Similarly, in their reformulated model of Schema Therapy theory, Arntz et al. (44) were able to derive 63 possible modes by combining 21 different early maladaptive schemas with 3 different coping styles. For simplicity’s sake, they reduced this number to *only* 40 modes. From the perspective of classification, how is the field to reconcile this proliferation of modes from different sources and methods? Even more importantly, how is the poor clinician to make sense of this buzzing confusion, given the need for a simple, heuristic mode model to guide clinical practice?

To resolve these issues, we believe that the field needs to start by agreeing on what modes are. Then, the various modes described in the literature can be tested against this definition.

Recently, Lazarus and Rafaeli (16) proposed a definition of modes as distinct and cohesive personality states, “gestalts” that consist of 4 components: affects, cognitions, behaviors, and desires (i.e., motivations), which tend to be coactivated at a given time. Applying this definition to the multiplicity of proposed modes, it is likely that some of them do not meet this complete definition, at least as far as they are currently specified. Part of this confusion arises from the tendency to equate modes with behaviors. For example, complaining or acting like a victim are simply behaviors, unless they are accompanied by the other characteristics that make for a coherent and distinct motivational state. In our view, modes descriptions must extend beyond labeling a behavior. A related issue is the need to determine when modes are distinct from one another. Some of the modes described in the literature show considerable overlap, and may represent different variations on the same mode, rather than distinct ones. Where and how to apply Occam’s razor will be essential moving forward to avoid a needless proliferation of modes. It will take time for the field to sort out these issues in classification. Careful clinical observations, theoretical developments, and empirical studies will all contribute to resolving them. In the meantime, we advise clinicians to take the mode descriptions we have offered not as “truths,” but as hypotheses that they can test in their own clinical work. To what degree do they find these mode descriptions illuminating, casting a light on phenomena that they have otherwise found confusing or difficult? Do these pseudo vulnerable modes, and our suggestions for intervening with them, help them to overcome obstacles in therapy? If so, then they pass the test of Young’s (10) original reason for introducing the mode concept, namely to guide intervention when emotional states present challenges for treatment.

## Conclusion

Pseudo vulnerability is an overlooked phenomenon in psychotherapy. These pseudo vulnerable coping modes interfere with the therapist’s usual attempts to intervene, leaving the client stuck in recurring self-defeating patterns, and the therapist increasingly frustrated and helpless. When psychotherapists inadvertently confuse pseudo vulnerability for authentic vulnerability, it may hinder the development of healthy individuation and coping while reinforcing outdated maladaptive interpersonal dynamics.

When working with pseudo vulnerable modes, it is essential for the therapist to keep in mind that *limited* reparenting must always include a balance of both care and manageable doses of frustration. Without care, the client cannot learn to operate from a healthy attachment perspective. Without frustration the client cannot develop a sense of resilience or independence. A reparenting recipe that provides a healthy balance between these two competing needs provides opportunities for the client to learn to tolerate uncomfortable feelings and the natural endings that are essential for operating in the world as a healthy adult, and to take the risk of learning to express vulnerability in more direct and authentic ways.

Given the complexity and often covert manifestation of pseudo vulnerable modes, access to ongoing supervision is key to identifying and differentiating them from authentic vulnerability, and choosing appropriate steps to address them. Where pseudo vulnerable modes run in sequence, therapists must prepare themselves for empathic confrontation which is compassionate but persistent, sticking to their methods while maintaining an awareness of their own schema activations. The process of supervision is also key to avoiding countertransference pitfalls, and the trap of the therapist defaulting to their own Self-Sacrifice/Rescuer mode. Each of these coping modes utilizes their own idiosyncratic communication style, and the more that therapists can familiarize themselves with these, the greater their capacity to address them in order to re-establish authentic connection.

## Data availability statement

The original contributions presented in the study are included in the article/supplementary material, further inquiries can be directed to the corresponding author.

## Author contributions

All authors listed have made a substantial, direct, and intellectual contribution to the work and approved it for publication.
